# The i-ACT™ in Obesity educational intervention: a pilot study on improving Canadian family physician care in obesity medicine

**DOI:** 10.1186/s12875-022-01715-w

**Published:** 2022-05-02

**Authors:** Sean Wharton, David Macklin, Marie-Philippe Morin, Jessica Blavignac, Stuart Menzies, Laura Garofalo, Michelle A. Francisco, Carol Thomas, Maxime Barakat

**Affiliations:** 1 McMaster University, Hamilton, Ontario Canada; 2 Department of Family and Community Medicine, Faculty of Medicine, University of Toronto, Toronto, Ontario Canada; 3 Institut universitaire de cardiologie et de pneumologie de Québec, Université Laval, Laval, Québec Canada; 4 Bausch Health Canada, Inc, Laval, Québec Canada; 5 CTC Communications Corporation, ON Mississauga, Canada; 6 Deca Medical Writing, London, Ontario Canada

**Keywords:** Obesity, Obesity management, Physicians, Family medicine, Medical education, Learning

## Abstract

**Background:**

Obesity is a chronic problem in Canada and although the Canadian Medical Association recognizes obesity as a disease, health care professionals (HCPs) are not necessarily proactively managing it as one. This study aimed to assess current obesity management knowledge and practices of Canadian family physicians (FPs) and evaluate the feasibility of an online self-directed learning platform, *i-ACT™ in Obesity,* in delivering learning and changing practice intentions to advance obesity management.

**Methods:**

i-ACT™ in Obesity is an online self-directed learning program designed by Canadian obesity medicine experts to provide individualized learning curricula to participants. One hundred FPs, with an interest in weight management and managing patients with obesity, were recruited across Canada to participate in a pilot study. FP education was delivered in a stepwise manner. Each participant completed a practice profile assessment to determine knowledge gaps and educational needs. Learners then watched didactic videos across disciplines on topics assigned to their curriculum by the program algorithm based on the relative difference between indicated and desired current knowledge. FPs also completed 10 retrospective patient assessments to assess clinical management practices and planned behaviour change. Feasibility, acceptability, and satisfaction of the learning program were assessed to formulate the rationale for a more widespread deployment in the future. Survey responses and related data were analyzed using comparative measures and descriptive statistics.

**Results:**

The program was piloted by ninety-one Canadian FPs, where 900 patients were assessed. FPs showed distinct differences between their current and desired levels of comfort in a variety of obesity-related topics. Participation was associated with an intention to use more obesity treatment interventions moving forward. The program received an overall satisfaction rating of 8.6 out of 10 and 100% of the evaluators indicated that they would recommend it to their colleagues.

**Conclusion:**

The program was overall well received and successfully changed obesity management intentions among participating FPs, thus setting the stage for a larger more comprehensive study to examine the efficacy of i-ACT™ in Obesity in addressing knowledge gaps and advancing evidence-based, guidelines-aligned approach to obesity treatment.

**Supplementary Information:**

The online version contains supplementary material available at 10.1186/s12875-022-01715-w.

## Background

Obesity has remained a chronic disease in Canada and elsewhere in the world for decades. According to the World Health Organization, obesity affects more than 650 million adults worldwide [1], while in Canada, about 7.3 million adults—about a fifth of the Canadian population—are currently classified as having obesity (body-mass index [BMI] ≥ 30 kg/m2) [2]. Direct costs associated with overweight and obesity were estimated in 2010 to represent 4.1% of total Canadian health care expenditures [3]. The percentage of Canadians classified as having obesity increased every year between 2009 and 2014 [4].

Unfortunately, despite these alarming numbers and the recognition by the Canadian Medical Association of obesity as a disease [5], health care professionals (HCPs) in Canada and elsewhere are not necessarily proactively managing this chronic disease, presenting a challenge for patients with obesity. For example, an American study noted that weight management counselling in primary care decreased between 2008-09 and 2012-13 [6]. The 2011 State of Obesity Care in Canada Evaluation Registry (SOCCER) study found that many Canadian HCPs did not raise the issue of weight with their patients affected by overweight and obesity [7]; similarly, a 2012 Canadian survey found that only 30% of patients with overweight or obesity were advised on weight management if they did not specifically request it [8].

Currently, the majority of Canadian HCPs do believe they have a role to play in this arena. The Canadian Awareness, Care and Treatment In Obesity MaNagement (ACTION) study, published in 2019, surveyed 2,000 people with obesity, 395 HCPs, and 150 employers on perceptions, attitudes, and perceived barriers to obesity management [9]. Although only 21% of people with obesity agreed that it was their HCP’s responsibility to actively contribute to their weight management efforts, 74% of HCPs surveyed agreed that they have a responsibility to help people with obesity to manage their weight.

This does not mean that Canadian patients are necessarily getting the help they need. Although HCPs in the ACTION study reported discussing weight with 72% of their patients in need of weight management, only 54% of the people with obesity surveyed reported having such discussions in the previous five years. Of those individuals who did report discussing weight, only 48% reported getting an obesity diagnosis from a qualified HCP, and only 28% reported having a weight-related call or follow-up scheduled. Moreover, although the 2020 Canadian guidelines recommend three pillars of obesity medicine: pharmacotherapy, psychological interventions, and bariatric surgery [10], in the SOCCER study, Canadian physicians did not recommend any single anti-obesity management strategy in more than 50% of their patients [7]. These studies did not suggest nor develop tools for improving physicians’ involvement in or knowledge about weight management, although it has been established that educational interventions for physicians and patients can make a difference in this field [11, 12].

The purpose of this study was to assess current obesity management knowledge and practices of Canadian family physicians (FPs) and evaluate the feasibility of using an online self-directed learning platform to deliver learning and change practice intentions. For this purpose, we developed a comprehensive online education platform called *Integrated Approaches to Change Treatment in Obesity (i-ACT™ in Obesity)* designed to evoke behaviour change and advance the clinical management of people with obesity. i-ACT™ in Obesity is an involved-learning platform developed to empower Canadian FPs with the knowledge and practical skills needed to improve the health outcomes of patients with obesity. In this pilot study we assessed the feasibility, acceptability, and satisfaction of the learning program for a more widespread deployment in the future.

## Methods

### Ethics approval and consent to participate

The Tri-Council Policy Statement (TCPS2) is a Canadian guideline for the ethical conduct of research involving humans and/or human biological materials. TCPS2 article 2.4 specifies that "REB review is not required for research that relies exclusively on secondary use of anonymous information, or anonymous human biological materials, so long as the process of data linkage or recording or dissemination of results does not generate identifiable information”.

A formal ethics committee review was not sought nor required for this program as it was developed for educational purposes only and was not intended to measure therapeutic outcomes. Participating FPs collected patient information through electronic medical records and chart audits to identify trends for self-reflection and educational purposes only. No identifying patient information was collected, and all FP- and patient-related data collected from the surveys were anonymized, prior to use in the study and is considered secondary use. Participants provided their consent to participate in the program by signing a participant agreement form.

Data were collected and analyzed in aggregate and were not linked to individual participant survey responses or consent information. The data analyzed in this manuscript is based solely on the use of the anonymized data and the results presented does not generate identifiable information. The manuscript was prepared in accordance with the Consolidated Standards of Reporting Trials (CONSORT) 2010 Checklist for pilot or feasibility studies.

### Design and setting

Although clinical practice guidelines are invaluable for bridging the gap between clinical practice and scientific evidence, guidelines are often poorly implemented into physician practices. Physician adherence is often limited by lack of awareness, lack of familiarity, and low self-efficacy. This results in a discrepancy between guideline recommendations and physician behavior. Knowledge Transfer Initiatives such as the Head to Heart initiative have been successfully used to elicit point-of-care knowledge transfer and facilitate guideline adherence [13]. We, therefore, set out to design an educational program to bridge the gap between obesity treatment guideline recommendations and clinical practice. The program was sponsored by Bausch Health Canada, and the continuing medical education company CTC Communication Corporation managed content development with the planning committee directly. Four Canadian experts in obesity medicine were identified as planning committee members: an endocrinologist, two internal medicine specialists, and one FP. Since educational topics in obesity are diverse and physicians’ learning requirements differ, the planning committee determined that the program should be individualized to the learner’s needs. They therefore designed a program to deliver education in a stepwise approach that included numerous touchpoints (Supplementary Fig. 1; Table 1) that involved both self-teaching and interactions with mentors and obesity experts. The pilot phase of the project invited 100 Canadian FPs to determine the feasibility, acceptability, and satisfaction of the program with an expansion planned after evaluation of the pilot.

**Table 1:** Linear steps involved in education delivery in i-ACT™ in Obesity

Interaction	Purpose
Physician Profile	• Understand the learners’ practice patterns • Determine their knowledge levels and their perceived knowledge gaps to provide individualized curriculum recommendations
Educational Videos	• Update the learner’s knowledge based on the specific needs identified in the *Physician Profile* • Learners watched up to 11 didactic videos across disciplines on topics from the health consequences of obesity to exercise recommendations and nutritional advice (Supplementary file 1) • The program algorithm assigned videos to a learner’s curriculum based on the relative difference between indicated desired and current knowledge on a variety of topics (Supplementary file 2)
Patient Assessments	• Learners were asked to identify 10 patients in their practice with obesity (BMI ≥ 30 or ≥ 27 with an obesity-related comorbidity) to assess their actual practice patterns in comparison to their perception
Obesity Plan of Action	• Allow for self-reflection of individual practice (based on the 10 *Patient Assessments*) compared to the aggregate of data from participants across the country • Provide feedback on management practices was provided from the Obesity Medicine Education Collaborative (OMEC) competencies [14]
Focus Group Meetings	• Small virtual group learning sessions were held between participants and Canadian obesity experts to allow for mentorship opportunities to support behaviour change

### Program operation and evaluation

Between August 2019 and August 2020, Canadian FPs were actively recruited (via e-mail and postcard invitations) to participate. FPs were identified and recruited based on their interest in obesity management and participation in previous obesity management-related programs and initiatives. Recruited FPs included those with interest in weight management, those currently active or not active in managing patients with obesity, and FPs representative of all key regions across Canada. Out of 100 invited FPs, 91 enrolled to participate in the study. After enrollment, education was delivered in a stepwise approach as shown in Table 1.

To collect baseline characteristics on FP knowledge gaps and to determine educational needs, practice profile surveys were developed and administered to FPs via the i-ACT™ in Obesity platform. To assess current management practices and planned changes to patient management, participating FPs also completed a patient assessment survey, which was a self-directed retrospective patient chart review for 10 of their patients with obesity. To be considered for the chart review, FPs were required to select patients aged 18 years or older, who had a BMI ≥30 kg/m2 or a BMI ≥27 kg/m2 with obesity-related comorbidities. Participating FPs retrieved patient information through electronic medical records and chart audits. Deidentified patient information was entered by the participating FPs into the platform and aggregated data was obtained for analysis. Prior to survey and platform launch, user acceptance testing was conducted and assessed through platform review and testing by the steering committee members.

After the program ended, 36 out of 91 participants voluntarily evaluated the five elements of the program (practice profile, educational videos, patient assessments, plan of action and focus group) in terms of what they added to the participant’s education experience. Program elements were evaluated quantitatively through ratings and qualitatively through open ended questions. Evaluators were also asked to rate the program overall and whether they would recommend it to a colleague.

### Data and statistical analysis

Data were collected from August 2019 to October 2020 and analysis was conducted using Microsoft Excel: demographic characteristics (FPs and patients), levels of comfort in discussing obesity topics (current vs. desired), and changes in obesity management interventions use (first vs. second self-assessment response) were analyzed using comparative measures. For dichotomous (yes/no) questions, the proportion of respondents who answered “Yes” were examined. For Likert-type questions (scored from 1 [strongly disagree] to 5 [strongly agree] or 1 [very poor] to 5 [excellent]), the weighted average for each ranking was calculated. Survey responses using standard descriptive statistics in Microsoft Excel were examined. Descriptive statistics such as mean, percentage, distribution, and range were analyzed by CTC Communications Corporation and reviewed by the planning committee.

## Results

### Primary results

**Fig. 1:**
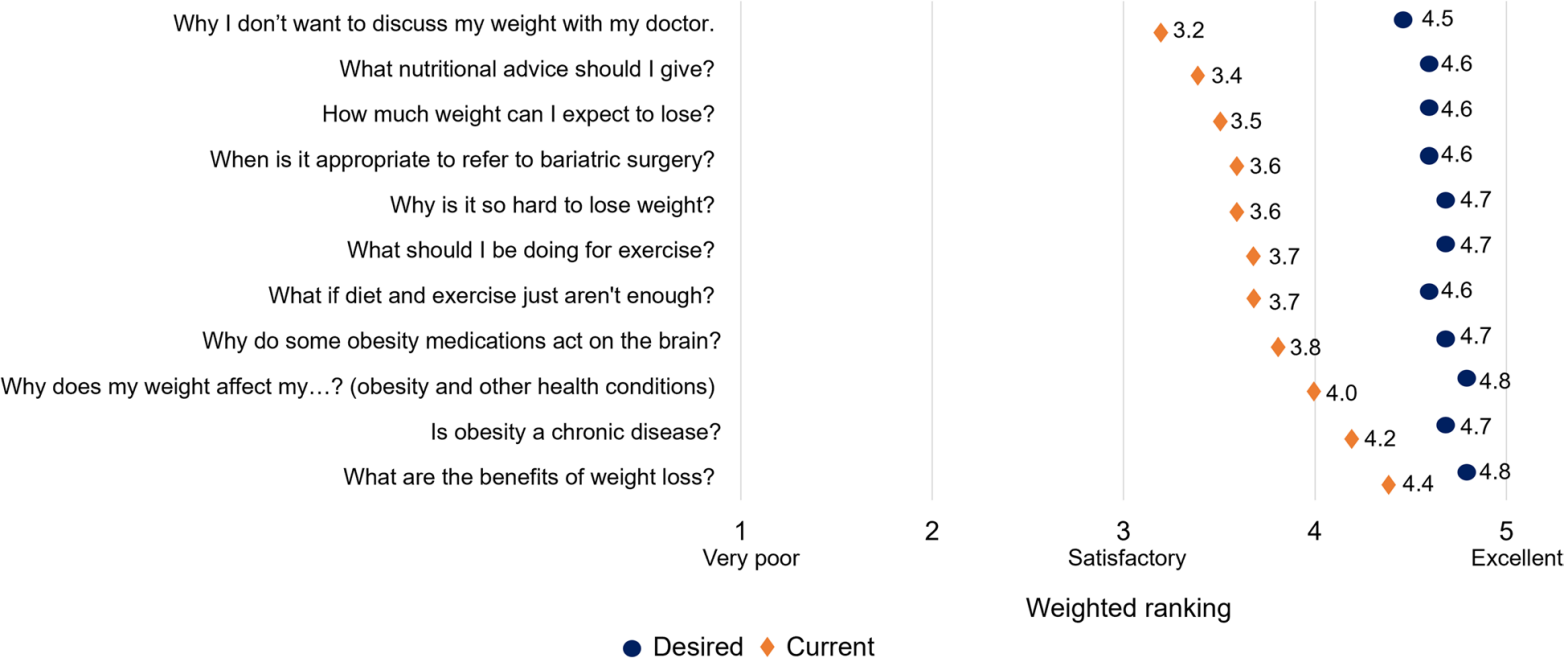
Current versus desired levels of comfort in discussing obesity topics with patients as reported during the baseline needs assessment (*N* = 91)

Ninety-one practice and 900 patient profiles were completed by FPs from across Canada (Table 2) between August 2019 and October 2020. There were distinct differences (as shown by differences on a five-point Likert scale) between the FPs’ current and desired levels of comfort with obesity-related topics frequently discussed with their patients (Fig. 1), demonstrating the need for education around this subject.

**Table 2:** Family physician characteristics

Characteristic	Percentage of FPs (***N*** = 91)
Region^a^	
Ontario	41%
Quebec	16%
Eastern Canada	9%
Western Canada	33%
Practice type	
Solo	39%
Group	56%
Hospital	2%
Academic	2%
Other	1%
Years in practice	
Less than 5	8%
5–10	12%
11–15	16%
16–25	18%
26–35	26%
More than 35	20%

^a^Eastern: FPs from Newfoundland and Labrador and Nova Scotia; Western: FPs from Alberta, British Columbia, and Manitoba.

### Family physician profiles before intervention

Prior to completing the i-ACT*™* in Obesity educational intervention, FPs did not consistently address weight loss with their patients with obesity. When a patient with overweight presented to their practice, 29% of FPs “always” and 54% “often” actively approached the topic of weight, while 16% of FPs “sometimes” and 1% “rarely” approached the topic. No FP indicated they “never” approached the topic of weight.

Interventions recommended to patients with obesity by FPs tended to focus on the “eat less and exercise more” approach. Eighty-six percent of FPs often recommended increased physical activity for obesity management, 60% often recommended calorie reduction, 57% often recommended a specific diet, and 32% often recommended the use of a food journal.

### Patient profiles

In the patients (Table 3) assessed in this program, comorbidities related to obesity were common, reported by 90% of patients (*n* = 810). Of those patients, 67% had hypertension, 65% had type 2 diabetes, and 88% had dyslipidemia. Of the patients with one or more comorbidities, 74% were experiencing depression and 64% were experiencing anxiety.

**Table 3:** Patient characteristics

Characteristic	(***N*** = 900)
Proportion female	63%
Mean age (years)	49.1
Mean BMI (kg/m^2^)	36.4
Comorbidities (mean)	1.8 per patient
Cardiovascular *(N = 449)*	50%
Hypertension	67%
Dyslipidemia	88%
Major adverse cardiovascular events	8%
Metabolic *(N = 442)*	49%
Diabetes	65%
Polycystic ovary syndrome [PCOS],	14%
Non-alcoholic fatty liver disease [NAFLD]	31%
Gout	7%
Hypothyroidism	20%
Mental health *(N = 292)*	33%
Depression	74%
Anxiety	64%
Binge-eating disorder	20%
Attention deficit–hyperactivity disorder [ADHD]	9%
Other obesity-related comorbidities *(N = 373)*	41%
Osteoarthritis [OA]	63%
Obstructive sleep apnea [OSA]	42%
Gastroesophageal reflux disease [GERD]	43%
Urinary issues	10%
None	10%
Reason for appointment	
Obesity-related complication	38%
Focus on weight	33%
Acute condition	19%
Other	10%

FPs reported that 91% of their patients understood the importance of weight reduction, but only 66% of patients had a very good or satisfactory knowledge of obesity. FPs indicated that they explained the concept of obesity as a chronic disease to 88% of their patients, however, only 63% of their patients understood this concept.

The interventions for weight management previously used by these patients were primarily focused on diet and exercise. Forty-seven percent of patients had tried dietary changes, 37% had tried physical activity changes, and 12% had tried pharmacotherapy. Of the 108 patients who had tried pharmacotherapy, 44% had tried liraglutide, 27% had tried naltrexone/bupropion, 15% had tried orlistat, and 12% had tried other weight management pharmacotherapy.

Patients reported significant barriers to an obesity management plan. Those most frequently cited included lack of adherence (20%), lack of support (12%), lifestyle changes being too restrictive (14%), dislike for lifestyle changes (13%), lack of time (13%), and cost of treatment (10%).

**Fig. 2:**
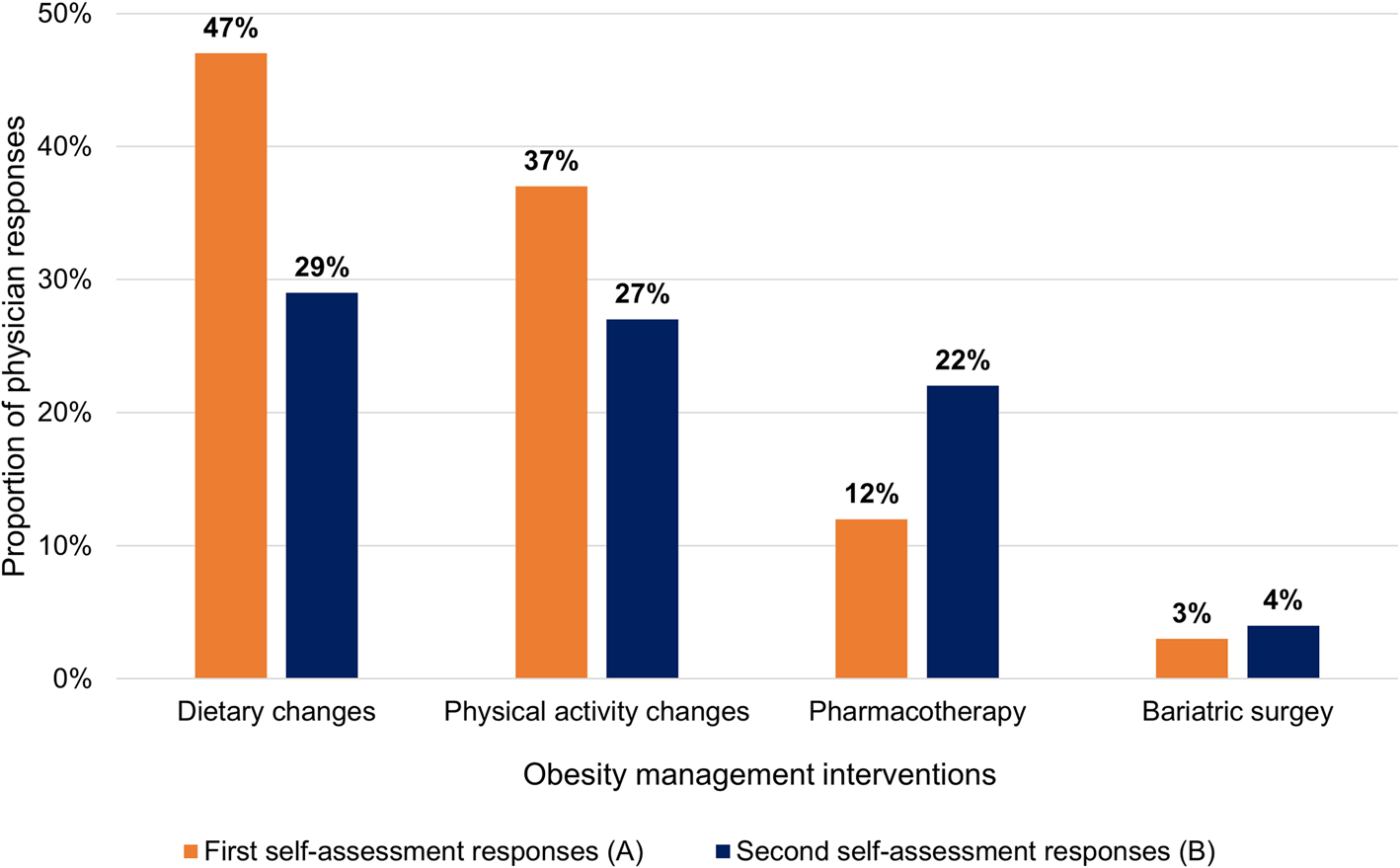
Changes in intervention use. **A** Responses to “What interventions have you ever used with your patient?” (first self-assessment, *N* = 91); **B** Responses to “What interventions are you considering today?” (second self-assessment, *N* = 900)

FPs recommended a wide variety of interventions to help their patients lose weight (Fig. 2). Weight management plans incorporated dietary changes (29%), physical activity (27%), pharmacotherapy (22%), referral to a dietitian and/or kinesiologist (15%), referral for bariatric consult (4%), and other interventions (2%).

When pharmacotherapy was recommended (in 198 patients), 59% of patients were recommended naltrexone/bupropion and 38% were recommended liraglutide. Of the patients referred for a bariatric consult, 52% had a BMI ≥ 40 kg/m^2^ (the recommended minimum BMI for bariatric surgery).

### Changes in FP intentions in providing obesity-related care

Participation in the i-ACT™ in Obesity program was associated with the intention to use more obesity treatment interventions, including pharmacotherapy, dietary and physical changes, and bariatric surgery referral. As part of the patient assessment step, FPs reported a mean of 2.0 interventions each patient had previously tried for obesity. When asked if they intended to make any changes moving forward, FPs indicated they intended to use a mean of 2.7 interventions. Participation was also associated with the intension to recommend fewer dietary changes and more pharmacotherapy and referrals (Fig. 2). As a result of the i-ACT™ in Obesity program, almost a quarter of participating FPs’ patients will now be considered for pharmacotherapy.

### Program satisfaction evaluation

**Fig. 3.**
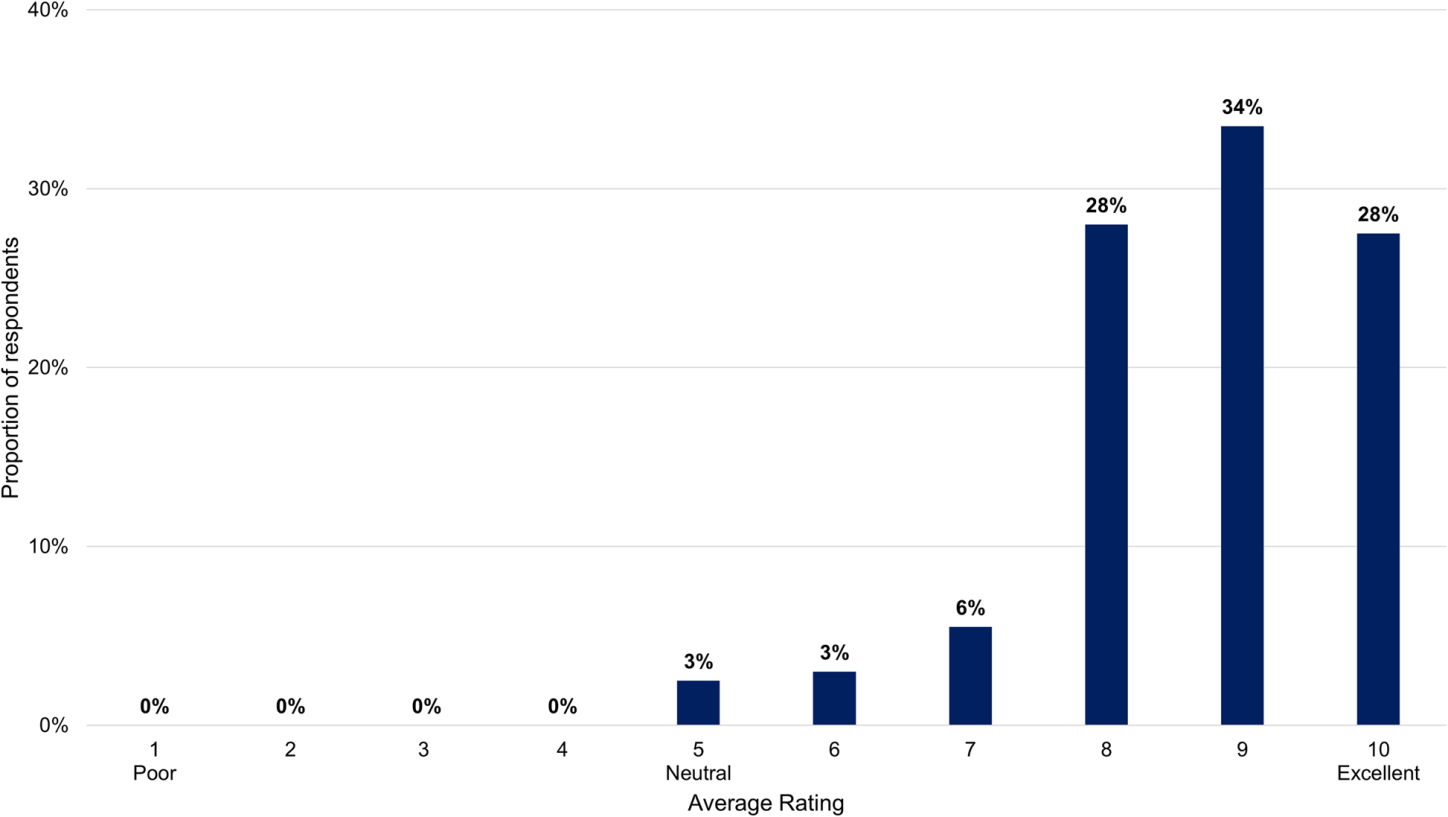
FP satisfaction with the i-ACT™ in Obesity program. Response to “How would you rate the program overall?” (Rate from 1 to 10, where 1= Poor, 5 = Neutral, and 10 = Excellent; *N*=36).

Out of the 91 FPs who participated in the study, 36 voluntarily completed the program satisfaction evaluation component. Rated on a Likert scale from 1 to 5 (where 5 meant “strongly agree that this element added value to my educational experience”), the five elements of the program scored between 4.2 and 4.7. Overall, the program was rated at 8.6 points out of a possible 10 (where 1 meant “poor” and 10 meant “excellent”) (Fig. 3). All 36 evaluators said that they would recommend the program to a colleague.

## Discussion

This pilot study aimed to assess current obesity management knowledge and practices of Canadian FPs and evaluate the feasibility, acceptability, and satisfaction of the i-ACT™ in Obesity educational program, in delivering learning and changing practice intentions to advance obesity management. Here, we identified a gap between current FP knowledge and the desired knowledge required for effectively managing patients with obesity. The i-ACT™ in Obesity learning program was well received and successfully changed obesity management intentions among participating FPs, indicating further research is warranted. This pilot study provided encouraging feasibility and satisfaction outcomes which can be used as a framework for a larger randomized controlled trial (RCT) efficacy study in the future.

The i-ACT™ in Obesity is a learning platform developed to provide individualized learning curricula to FPs by facilitating involved learning and self-reflection while: 1) improving the understanding of obesity as a chronic pathology and its impact on the patient’s journey, 2) advancing management of patients with obesity in Canadian primary care, and 3) providing opportunities for collaboration with peers and expert mentors. Canadian FPs showed distinct differences between their current and desired levels of comfort with a variety of obesity-related topics frequently discussed with patients. Before the program intervention, participating FPs tended to focus on the “eat less and exercise more” approach as their management strategy for patients with obesity. After the intervention, participants changed their intentions toward obesity management, intending to recommend fewer dietary changes and more pharmacotherapy and specialist referrals.

Forty-four percent of participating FPs reported in their original self-assessments that they recommended antiobesity medications often or very often, and 12% of the patients seen by these FPs had already tried pharmacotherapy for weight management. This suggests that the participants in i-ACT™ in Obesity began the program as unusually high users of obesity-targeted pharmacotherapy. By contrast, only 12% of HCPs in the 2019 ACTION Canada study reported discussing prescription medication with their patients with obesity [9]. This difference may be accounted for by the fact that the i-ACT™ in Obesity participants were selected for their interest in obesity management and likely volunteered to participate due to their interest in this topic.

Despite being developed before the publication of the new Canadian obesity guidelines, the i-ACT™ in Obesity intervention appears to have notably changed obesity management intentions among participating FPs, moving them away from an “eat less and exercise” approach and toward a more evidence-based, guidelines-aligned approach involving pharmacotherapy and referrals to dietitians, kinesiologists, and bariatric specialists (10). The i-ACT™ in Obesity platform is now available to physicians alongside the guidelines, providing a wider range of options for increased learning and confidence.

It has been shown in fields other than obesity that education of HCPs improves their knowledge, confidence, and enthusiasm for management of specific conditions [15]. Multimedia- or Internet-based educational interventions have been shown to improve surgical performance [16] and the detection of child abuse by nurses [17]. Systematic reviews have shown that Web-based learning improved nurses’ knowledge and skills performance [18], and computer-assisted learning improved knowledge gain in orthodontic students [19]. Here we demonstrated the feasibility, acceptability, and satisfaction of the i-ACT™ in Obesity program in furthering FP knowledge of obesity management guidelines and changing clinical care intentions. We hope that i-ACT™ in Obesity will join a growing list of interventions aimed at disseminating important knowledge and skills in the field of obesity management. Our intention is to deploy this program to a wider range of physicians and evaluate its efficacy in driving behavioural changes in physicians as well as patients when it comes to obesity care and management.

### Limitations

This educational intervention was conducted as a pilot study and due to the relatively small sample size of the participants, the characteristics, responses, and behaviour of the respondents may not be representative of the majority of Canadian FPs. Although our results appear to have notably changed obesity management intentions among participating physicians, a larger-scale study with more physicians (and patients) is needed to assess generalizability. It may also be valuable to include a follow-up period to assess practice changes following the program intervention to determine whether the planned changes were implemented. Moreover, this study utilized only one group with no comparator arm. Future improvements could include a RCT (comparing a control group with no intervention to one receiving the i-ACT™ in Obesity educational intervention) as well as a follow-up survey to determine efficacy, and impact on physician behaviours as well as an assessment of patient success.

There was some misalignment noted around FP explanations and patient beliefs—such as the difference seen between FPs’ communication that obesity is a chronic disease and their perception of patients’ understanding of that fact—perhaps suggesting that not all patients have confidence in their FPs’ explanations. Patient-reported characteristics, preferences, satisfaction, and health -outcomes were not considered in this pilot phase. Although clinical management of the patients may not differ significantly before or after the educational intervention, this possibility cannot be excluded. It may be of interest to include a self-reported patient assessment to the program to further address these differences.

Differences in FPs’ comfort levels with the various topics also suggested the existence of biases and possible paternalism, since the group perceived themselves to be very comfortable discussing the benefits of weight loss—something that physicians might lecture their patients about—but much less comfortable talking about the reasons patients do not want to discuss their weight with their doctor. Future research could explore whether there are additional evidence-informed strategies that would encourage more physicians to recognize and mitigate these biases.

## Conclusions

i-ACT^TM^ in Obesity is a comprehensive platform that delivers obesity education across a diverse range of topics, with educational interventions and content specific to a participating physician’s learning needs. It has demonstrated the ability to alter FPs’ intentions toward more evidence-based management of obesity, and we believe it may be used long-term as a valuable educational tool. We hope that use of this platform by additional physicians will both improve currently limited levels of physician knowledge and provide further data about Canadian family physician management of patients with obesity.

## Supplementary Information

**Additional file 1: Supplementary Figure 1.** Participants of i-ACT™ in Obesity.

**Additional file 2: Supplementary file 1.** Titles and learning objectives of 11 didactic educational videos included in i-ACT™ in Obesity.

**Additional file 3: Supplementary file 2.** Algorithm for determining learner’s i-ACT™ in Obesity video curriculum.

## Data Availability

Anonymized datasets used and analyzed during the current study may be available upon reasonable request to the corresponding author.

## Abbreviations

ACTION: Awareness, Care and Treatment In Obesity MaNagement
ADHD: Attention deficit–hyperactivity disorder
BMI: Body-mass index
CONSORT: Consolidated Standards of Reporting Trials
FP: Family physician
GERD: Gastroesophageal reflux disease
HCP: Health care professionals
i-ACT™: Integrated Approaches to Change Treatment
NAFLD: Non-alcoholic fatty liver disease
OA: Osteoarthritis
OMEC: Obesity Medicine Education Collaborative
OSA: Obstructive sleep apnea
PCOS: Polycystic ovary syndrome
RCT: Randomized controlled trial
SOCCER: State of Obesity Care in Canada Evaluation Registry
