# Supplementary figures and images for: The i-ACT™ in Obesity educational intervention: a pilot study on improving Canadian family physician care in obesity medicine
Source: BMC Prim Care. 2022 May 2;23:101. doi: 10.1186/s12875-022-01715-w (PMC9059350; doi:10.1186/s12875-022-01715-w)

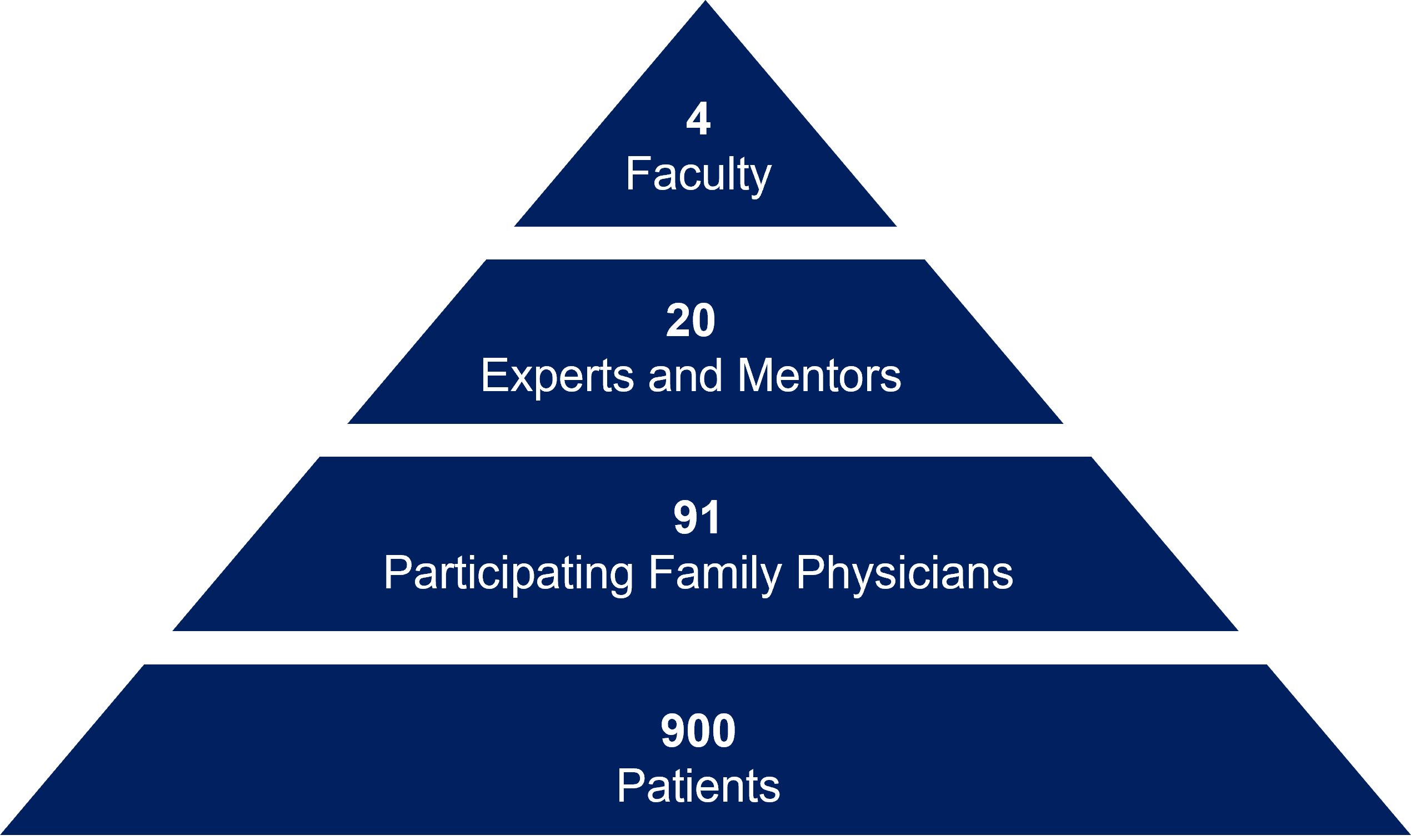

Supplement: Supplementary file 1 — Additional file 1: Supplementary Figure 1. Participants of i-ACT™ in Obesity. [file 12875_2022_1715_MOESM1_ESM.tif]
