# Supplementary material for: The i-ACT™ in Obesity educational intervention: a pilot study on improving Canadian family physician care in obesity medicine
Source: BMC Prim Care. 2022 May 2;23:101. doi: 10.1186/s12875-022-01715-w (PMC9059350; doi:10.1186/s12875-022-01715-w)
Supplement: Supplementary file 2 — Additional file 2: Supplementary file 1. Titles and learning objectives of 11 didactic educational videos included in i-ACT™ in Obesity. [file 12875_2022_1715_MOESM2_ESM.docx]

**Supplementary File 1. Titles and learning objectives of 11 didactic educational videos included in i-ACT™ in Obesity.**

| **Video Title** | **Learning Objectives** |
| --- | --- |
| 1. Is Obesity a Chronic Disease? | - Review the rationale for classifying obesity as a chronic disease - Peripheral controls of energy balance - Etiology of obesity is multifactorial - Why weight management requires long-term maintenance - The clinical treatment options for patients with obesity |
| 1. Why Is It so Hard to Lose Weight? | - Review the physiologic, hedonic, and cognitive controls of energy intake - Determine the impact of weight reduction on these controls - Understand that the body is designed to defend against weight loss |
| 1. What Are the Health Consequences of Obesity, and What Are the Benefits of Weight Loss? | - Review the most common obesity-related co-occurring diseases - Review the impact of weight loss on obesity-related co-occurring diseases |
| 1. Why Does My Weight Affect My …? | - Review the guideline recommendations for weight management for many chronic conditions - Review the potential changes in the management of obesity since obesity guidelines were published |
| 1. Why I Don't Want to Discuss My Weight With My Doctor | - Review different strategies to approach the topic of weight reduction in a person living with obesity - Examine psychological strategies in a person interested in reducing weight - Discuss behavioural interventions for the management of obesity - Review behavioural interventions for overcoming expected and unexpected barriers |
| 1. What Should I Be Doing for Exercise? | - Understand that physical activity does not consistently lead to weight reduction without dietary intervention - Review the multiple health benefits of physical exercise, beyond weight reduction - Understand how to customize physical activity recommendations based on the patient, including the application of FITT principles |
| 1. What Nutrition Advice Should I Give? | - Review the biological and environmental factors leading to energy intake - Understand how to effectively provide patients with advice on nutrition and obesity management - Identify when to refer a patient to a registered dietitian for individualized nutrition care |
| 1. What if Diet and Exercise Just Aren’t Enough? | - Review Canadian pharmacotherapy options for weight management and identify candidate patients - Understand the mechanism of action for obesity pharmacotherapy - Review pharmacotherapy management and optimization in patients with obesity |
| 1. When Is It Appropriate to Refer to Bariatric Surgery? | - Identify patients for whom bariatric surgery would be the most effective obesity management option - Review the types of bariatric surgery and the health benefits of each type |
| 1. Mechanism of Action of Antiobesity Medications | - Understand how liraglutide and naltrexone/bupropion address the underlying neurobiology of obesity - Review the psychiatric medications associated with weight gain - Discuss how drug interactions can be managed with adjustments in doses and monitoring |
| 1. How Much Weight Can I Expect to Lose? | - Review the challenge of weight loss maintenance - Examine the weight loss effect of various obesity interventions - Introduce the concept of ”best weight” - Discuss strategies to support patients in maintaining lost weight and preventing weight regain |
