# Supplementary material for: The i-ACT™ in Obesity educational intervention: a pilot study on improving Canadian family physician care in obesity medicine
Source: BMC Prim Care. 2022 May 2;23:101. doi: 10.1186/s12875-022-01715-w (PMC9059350; doi:10.1186/s12875-022-01715-w)
Supplement: Supplementary file 3 — Additional file 3: Supplementary file 2. Algorithm for determining learner’s i-ACT™ in Obesity video curriculum. [file 12875_2022_1715_MOESM3_ESM.docx]

**Supplementary File 2. Algorithm for determining learner’s i-ACT™ in Obesity video curriculum.**

There are 11 didactic educational videos available to i-ACT™ in Obesity learners. Three videos were mandatory for each learner as determined by the Steering Committee: (1) *Why Is It so Hard to Lose Weight?* (2) *What if Diet and Exercise Just Aren't Enough?* and (3) *How Much Weight Can I Expect to Lose?*

The remaining eight videos were added to a learner’s curriculum by the program algorithm based on the relative difference between their indicated desired and current knowledge on eight obesity-related topics (Supplementary 1, #4–11). For each of these eight topics, learners were asked to rate their current knowledge and their desired knowledge on a five-point Likert scale, where values were assigned to each response option as follows: Excellent = 5, Very good = 4, Satisfactory = 3, Poor = 2, and Very Poor = 1. For each of these eight topics, the algorithm calculated the difference between learners’ desired knowledge and current knowledge, herein Δ. For any video topic in which Δ ≥ 1, the program’s algorithm added the corresponding video to the learners’ “recommended” video section, sorted by largest to smallest Δ. For any video topic in which Δ < 1 (including negative numbers), the program’s algorithm added the corresponding video to the learners’ “optional” section. Recommended and optional videos were not mandatory for program completion. Learners could select from the list of recommended and optional videos, and their selection would queue with the three mandatory videos.
